# Female university students’ preferences for different types of sexual relationships: implications for gender-based violence prevention programs and policies

**DOI:** 10.1186/s12905-020-01131-1

**Published:** 2020-11-30

**Authors:** Laura Ruiz-Eugenio, Sandra Racionero-Plaza, Elena Duque, Lidia Puigvert

**Affiliations:** 1 Department of Theory and History of Education, University of Barcelona, Barcelona, Spain; 2 Department of Sociology, University of Barcelona, Barcelona, Spain; 3 Affiliated member of the Centre for Community, Gender and Social Justice. Institute of Criminology, University of Cambridge, Cambridge, UK

**Keywords:** Dominant coercive discourse, Dating violence, Gender violence, Intervention and prevention programs, Young women, Risk factors, Social influence, Socialization

## Abstract

**Background:**

Gender-based violence among young women is a growing problem worldwide. The consequences of this victimization have been well reported in the scientific literature, among which negative health outcomes stand out. The factors influencing this problem are many; one highlighted by research is socialization into a dominant coercive discourse that associates sexual-affective attraction to males with violent attitudes and behaviors, while in turn, such discourse empties males with egalitarian behaviors from sexual attractiveness. This coercive discourse may be shaping the sexual preferences of female youth. The current paper explores young women’s preferences for different types of sexual relationships and, more particularly, for what type of sexual affective relationships they coercively preferred men with violent attitudes and behavior.

**Methods:**

A quantitative, mixed-design vignette study was conducted with 191 college females in Spain. We focused the analysis only on responses about vignettes including narratives of men with violent attitudes and behaviors. In addition, we examined whether participants would report higher coerced preferences for violent men when asked about the coerced preferences of their female friends than when asked about their own preferences.

**Results:**

Only 28.95% of participants responded that their female friends would prefer a young man with violent behavior for a stable relationship, meanwhile 58.42% would do it for hooking up. When reporting about themselves, the difference was greater: 28.42% would prefer a young man with violent behavior for hooking up and just 5.78% for a stable relationship.

**Conclusions:**

The dominant coercive discourse that links attractiveness to people with violent attitudes and behaviors may be explaining the results obtained in this study. The findings can help eliminate the stereotype largely adopted by some intervention and prevention programs which assume that gender-based violence occurs mainly in stable relationships, considering that falling in love is the reason that lead women to suffer from violence. Our results can also support health professionals and others serving young women to enhance their identification of gender violence victimization, as well as our findings point to the need to include the evidence of gender violence in sporadic relationships in prevention programs and campaigns addressed to young women.

## Background

The World Health Organization (WHO) stated in 2013 that overall, 35% of women worldwide have experienced either physical and/or sexual intimate partner violence or non-partner sexual violence [1]. Later, in 2014, the European Agency for Fundamental Rights released the ‘Violence against women: an European Union (EU)-wide survey’ report [2], stating that an estimated 13 million women in the EU had experienced physical violence in the course of 12 months before the survey interviews. The same year, the European Agency for Fundamental Rights [2] revealed that in relation to minors, one in three had experienced physical and or sexual violence since she was 15 years old, and out of all women with a (current or previous) partner, 22% had experienced physical and/or sexual violence by a partner since the age of 15. Regarding non-partner violence, 22% has experienced physical violence by someone other than their partner since the age of 15. This data reflects that gender violence is still a problem in the EU and worldwide [1], affecting an important number of young women, and having huge consequences on the victim and also on the social networks of both the abuser and the victim.

In the attempt to investigate the roots of this problem, previous studies evidence that one of the causes of the increasing numbers of gender-based violence victimization is found in the dominant discourse that coerces adolescents and youth and which links attractiveness to men with violent behaviors. This is known as *dominant coercive discourse* [3], the discourse which, shaped by an imbalance in power within relationships, influences socialization into linking attractiveness to people with violent attitudes and behaviors, while non-violent people and relationships are -because of this coercive dominant discourse- mostly perceived as convenient but not exciting. This model can be identified either in stable or sporadic relationships [4]. This line of research has shed light on the role played by social media, adolescent and youth literature, movies, TV series, etc., in teaching the coercive discourse [5–7]. As Gomez [5] demonstrates through an extensive analysis of young magazines, boys with violent behaviors and attitudes are presented as more attractive and sexually exciting; thus, passion is associated with risk. In so doing, this coercive discourse leaves less option to experience a relationship that is, at the same time, egalitarian and passionate and implies the separation between what is considered as convenient in the long run, and what is seen as passionate and exciting in the short run, a fracture which is reflected in the daily life of many youth [8]. Quoting the words of a 15-year-old girl from a teenage magazine, *Ragazza*: “My parents tell me to marry a good boy, and I really listen to them. Until I have to get married, I’m having fun with bad boys” (p.170).

Yet all women are able, if they freely choose to do so, to critically reflect upon the coercive discourse and, from such critical consciousness, make choices and develop preferences free from coercion. Women’s agency to counteract dominant and coercive discourses has been well documented throughout history in feminist literature [9]. Also, along the lines of agency and the topic discussed here, interventions of preventive socialization of gender violence, such as the dialogic feminist gatherings, implemented in the context of the Free Teen Desire research project, have well evidenced the importance of women being aware of and discussing the coercive discourse in order to counteract it and exercise their agency in deciding upon their preferences and partner selection, if the participant women freely choose to do so.

Studies conducted from the field of social psychology [10] shed light on how this dominant discourse operates. In an experiment, female participants responded that in relation to different descriptions regarding a soldier’s experience after coming back from war, their preference for the ‘warrior’ was higher when asked for short-term relationships against long-term relationships. In a different investigation [11], researchers observed that female individuals who declared themselves as wanting to avoid boredom and looking for exciting social activities preferred a dominant partner. Thus, those perceived as dominant were considered by that type of female participants as more interesting, attractive, funny, and appealing. Likewise, women who defined themselves as liking new and exciting social activities, such as parties, social drinking, and casual sex, also preferred a dominant partner [11]. Research in evolutionary psychology focused on effects of sexual dimorphism on facial attractiveness has provided complementary data. Studies on this topic [12] have shown that enhancing masculine facial characteristics increased both perceived dominance and negative attributions, such as coldness or dishonesty, being those attributions associated with participation in deceptive behavior in stable or long-term relationships and paternal investment.

This evidence is of concern from the point of view of violence against women. Data on prevalence of gender violence in dating relationships has been collected in the US for decades [13–15], and some specific surveys on adolescent dating violence (ADV) have been conducted in Europe [2, 16]. Nonetheless, existing figures on dating violence worldwide is limited. In a literature review conducted by Leen and colleagues [17], authors point out that the tradition of collecting data relating to ADV prevalence is not as well established in Europe as in North America, what according to them explains the scarcity of statistics in many European countries and the subsuming of dating violence measures within broader measures of peer violence (pg. 172). That is the case of Spain. For the case of the US, the US national survey *Youth Risk Behavior Surveillance System* [15] shows that the prevalence of physical dating violence among females was in 2013 of 13%, and that the prevalence of sexual dating violence was 14.4% [18]. Other important data are collected in the US National Survey on *Teen Relationships and Intimate Violence* for 12- to 18-year-olds focused on Adolescent Relationship Abuse (ARA) perpetration and victimization [19]. The results indicate that 37% report current or past-year dating and 69% report lifetime ARA victimization (63% lifetime ARA perpetration). Although psychological abuse was most common for these youths, the rates of sexual abuse victimization (18%), physical abuse victimization (18%), and physical abuse and/or sexual abuse perpetration (12%) are substantial. For the case of Germany, Brzank and colleagues [16] conducted a cross-section survey in 2012–2013 with 462 pupils aged between 14 and 17 aimed at capturing teen dating violence prevalence, risk factors and prevalence, in which indicated that 77% of them had had experiences with dating and intimate relationships [16]. This survey revealed that 66% of female and 60% of male persons at risk reported for at least one kind of Teen Dating Violence (TDV), and the most common type of TDV was emotional violence.

The consequences of teen and young-adult dating violence victimization and adverse health-outcomes have been largely documented. To fall victim of dating violence can be a precursor for intimate partner violence (IPV) victimization in adulthood, which is most notable among women, as they are more likely to engage in high-risk sexual behavior, unhealthy dieting behaviors, substance-and alcohol-abuse, depressive symptomatology, and suicidal ideation/attempts [20, 21]. The consequences are also adverse for the brain and other organs. Women who are victims of IPV may be characterized by alterations in hypothalamic-pituitary-adrenal axis functioning, which can manifest in anxiety disorders, digestive problems, fatigue, and insomnia among other symptoms [22]. Generally, victims of dating violence in adolescence are associated with self-reports of poor physical health and more concerns about health [13].

Although at the international level steps are being made in order to implement evidence-based programs for the prevention of both domestic violence and other types of gender-based-violence [23], in Spain, despite the existing evidence about dating violence, most of the publications at the national level and most of the gender violence prevention programs start from the premise that violence against women mainly happens in stable relationships. The same idea persisted at the legal level. The existing legislation, *The 2004 Spanish Act against Gender Violence* [24]*,* the first of its kind in Europe, did not take into account dating violence, but it solely acknowledged that gender violence is the one that is perpetrated against women by her partners or ex-partners. Consequently, most of the adolescent training in Spain for preventing gender violence is focused on questioning stable relationships and avoiding falling in love, associating and entangling both ideas (stable relationships = romantic idea of love or ideal love) and thus considering falling in love as a road to victimization [25, 26]. Thus, instead of pointing at the influence of the aforementioned coercive discourse in youth socialization processes, most discourses in the media, interventions and prevention programs in Spain indicate that is the notion of romantic ideal of love the cause of gender violence [26–30]. For example, some of those gender violence prevention programs addressed to female youth have employed the motto “Love kills”, which in Spanish (“El amor mata”) has about 85.000 entries in Google on May 14th, 2020. Such association between romantic/ideal love and violence against women also finds its expression in training programs in Spain addressed to teachers and psychologists.

There is no scientific evidence supporting that socializing into “ideal love”, −which is always free of violence, and doing so from diversity of options and individual freedom, causes gender violence [31]. The lack of solid scientific evidence in mainstream prevention campaigns about gender violence in Spain has serious consequences, especially for young women, who end up being victims, as these campaigns have as main goal to dismantle the conception of searching for the ideal of love, instead of informing about the risk factors that can lead to violence in any kind of relationship, and how the coercive discourse operates and shapes young women’s coerced preferences.

Summarizing, in Spain, there is no tradition of evidence-based campaigns to prevent gender violence, so it is important to be aware of: 1) While the international scientific community has shown that gender violence exists in both stable and sporadic relationships, prevention campaigns in Spain are based on the fact that gender violence occurs mainly in stable relationships, in the same way, the *2004 Spanish Act against Gender Violence* does not take dating violence into account; 2) prevention campaigns that have as their motto, for example, “love kills”, have been promoted instead of identifying that it is not love that kills but rather violent men; 3) as a consequence, the focus of theses campaigns has not been placed on stopping the dominant coercive discourse that associates attraction to men with violent attitudes and behavior. Therefore, in Spain, evidence-based gender violence prevention campaigns are needed to dismantle the assumptions that gender violence occurs mostly in stable relationships and that love kills, as well as to break down the dominant coercive discourse of attraction to violent men. Evidence-based prevention campaigns could be directed at promoting a discourse that shows the violent as undesirable, promoting that women could freely choose if they wish for their relationships, both sporadic and stable, non-violent men, thus contributing to reducing the risk of victimization of women.

This study aims to provide new evidence on how the dominant coercive discourse that associates men with violent behavior with attractiveness coerces the values, likes and preference of 191 young women in Spain, which is a risk factor for gender-based violence victimization.

### Current study

This study was part of experimental research carried out under the framework of the Free Teen Desire research project led by the University of Cambridge (United Kingdom) under the European Union’s Horizon 2020, Marie Sklodowska-Curie grant in 2015. In line with the preventive socialization of gender violence approach developed at the Community of Researchers on Excellence for All [32], the project’s main goal was to contribute to better understand how the coercive discourse which associates attractiveness with men with violent behavior and attitudes [5, 32] coerces young women’s values, likes and preferences, and how such coercion can later constitute a risk factor for violence victimization. This investigation was conducted in secondary schools in four European countries (UK, Spain, Finland and Cyprus) and in universities in Spain.

This article reports results from the study with female college students. In this investigation a quantitative, mixed-design vignette study was conducted in Spain in 2015 with 191 female university students to explore young women’s preferences for different types of sexual relationships, hooking up and stable relationships. In this examination as well as in the discussion of the findings, we considered results from prior research [5, 8] that indicate the power of the coercive discourse in sexual-affective socialization. This article solely discusses data about participants’ responses for men with violent attitudes and behavior. The reason for this choice is that this article focuses on the evidence showing how the dominant coercive discourse, which associates attraction to men with violent attitudes and behaviors, impacts the preferences of 191 young women in Spain. This decision has behind it the researchers concern about the impact on young women that the prevention campaigns for gender violence in Spain are not evidence-based and do not put the focus on identifying and rejecting these violent attitudes and behaviors in all types of relationships, both stable and sporadic. Other articles in progress put the focus on other factors that the Free Teen Desire research project has analyzed. Also, following the scientific literature on gender and masculinities [33], it was assumed in the study that not all dominant traditional masculinities or hegemonic masculinities are violent, yet all violent masculinities are dominant. In our study, the violent male behaviors and attitudes of dominant men presented to participants were illustrations of what the international scientific literature has detailed as constituting psychological, physical or sexual violence against women [34].

This research is undertaken at a promising moment. Since the end of 2016, the *2004 Spanish Act against Gender Violence* has been again in the public debate because of the reopened discussion about its mistake of ignoring violence perpetuated against women by other actors different that the partner or ex-partner. Steps are being made by different actors in order to achieve that the Spanish Parliament amend the *2004 Act* along the lines of widening its definition of what constitutes gender violence, in line with the Convention on the Elimination of all forms of Discrimination Against Women (CEDAW) and the Council of Europe Convention on preventing and combating violence against women and domestic violence (Istanbul Convention), ratified by Spain. This will involve considering gender violence in other domains, besides domestic violence, including dating violence. If this amendment materializes, it will be a crucial milestone in the fight against gender violence. At the time of writing this article that debate is stalled and there has not yet been any modification of the *2004 Spanish Act against Gender Violence.* Research data can be central to support and inform this change in the law, as well as to base in scientific evidence related policies and strategies both already in place and future ones that will result from the extension of what is understood as gender violence in the law. The research conducted in the Free Teen Desire research project, and reported partially in this article, is a contribution in this regard.

## Methods

### Participants

The participants included 191 first-year female students who were aged 18–29 years (mean age 19.78 years) studying a degree in education at two public Spanish universities. Among the respondents, we reported one missing case. All participants took part in the study on a voluntary basis. A total of 176 students (92.14%) reported European white heritage; 4 students (2.09%) reported Arabic heritage; 3 (1.57%) reported Latin heritage; 1 (0.52%) reported Roma heritage; 1 (0.52%) reported other cultural or ethnic origin, and 6 (3.14%) did not answer this question. This demographic composition was consistent with the lack of diversity in the Spanish institutions of higher education: for the period 2014–2015, the percentage of foreign students in Spanish universities was 4.01%.^16^

The study participants were divided into the following four groups: group 1 (*n* = 61), group 2 (*n* = 55), group 3 (*n* = 48), and group 4 (*n* = 27). These groups were natural, as they corresponded to the same four class groups that participated in the project.

### Instrument and measures

The study used an experimental vignette-based methodology (EVM) [35], an approach built on hypothetical situations that is commonly used in studies aimed at analyzing behaviors and conceptions about attractiveness and sexual attitudes in heterosexual adolescents and young adults [36, 37]. The sets of vignettes designed for the Free Teen Desire research project did not only used EVM to examine young women’s attraction and sexual-affective emotion and thoughts, but the project was also ground-breaking methodologically as it employed EVM to measure young women’s coerced preferences to have a ‘hook up’ or a stable relationship with young men with violent behavior. It was in the Free Teen Desire research project when EVM was used for the first time to undertake this examination through the Free Teen Desire questionnaire (see supplementary material 1 Free Teen Desire questionnaire), designed within the framework of the aforementioned project.

Following a mixed research design [38] three sets of vignettes were created (set A, set B, set C) combining a total of eight different pictures of young men and eight different narratives of male sexual affective behavior (see Table 1). From these combinations, a total of 12 different vignettes of young men’s profiles were ultimately designed.

**Table 1:** Composition of vignette sets

Vignette Set A		Vignette Set B		Vignette Set C	
*Violent behavior*					
Vignette 1	Vignette 3	Vignette 5	Vignette 7	Vignette 9	Vignette 11
Picture 1	Picture 2	Picture 5	Picture 6	Picture 3	Picture 4
Narrative 1	Narrative 2	Narrative 1	Narrative 2	Narrative 3	Narrative 4
*Non-violent behavior*					
Vignette 2	Vignette 4	Vignette 6	Vignette 8	Vignette 10	Vignette 12
Picture 5	Picture 6	Picture 1	Picture 2	Picture 7	Picture 8
Narrative 5	Narrative 6	Narrative 5	Narrative 6	Narrative 7	Narrative 8

Vignettes sets A, B and C were randomly distributed among the four student groups (g1, g2, g3, g4). Within each group, all participants had the same vignettes (see Table 2).

**Table 2:** Distribution of vignette sets among groups

Study participants group (g)	Vignette set
g1	A
g2	B
g3	C
g4	B

**Fig. 1:**
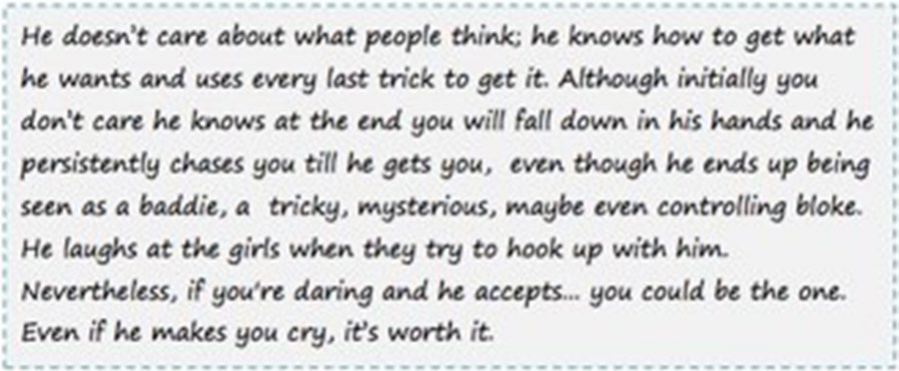
Example of a narrative describing a man’s violent attitudes and behavior

Each set included four different vignettes; each was based on a picture of a young man accompanied by a short narrative that described mostly his sexual-affective attitudes and behavior with women. In two of the vignettes, the narratives described the man’s behavior and attitudes as violent or sexist, according to the scientific literature [39–42]. Examples of descriptors provided in the narratives are the following: ‘show-off’, ‘controlling’, ‘manipulative’; ‘disdains’; ‘he ends up being seen as a baddie’, ‘a tricky’, ‘mysterious’, ‘maybe even controlling bloke’; ‘he has touched them without consent’ (see Fig. 1).

**Fig. 2:**
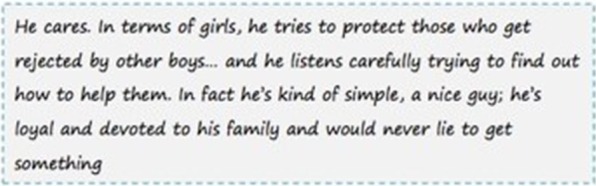
Example of a narrative describing a man’s non-violent attitudes and behavior

In the other two vignettes within each set, the narratives described the behavior and attitudes of the man in the picture as neither violent nor sexist, but rather as kind (see Fig. 2).

As shown in Table 1, the pictures among the violent and non-violent profiles were switched between sets A and B but the narratives were kept, so that the pictures that accompanied the narratives of violent behavior in set A became the pictures for the narratives of non-violent behavior in set B. This design supports examining the influential role of sexual-affective male behavior (violent or non-violent) beyond the image of the male. For vignette set C, other pictures and narratives were used.

Within each set, each of the four vignettes (picture and narrative combined) was followed by a short survey. In the first page of the vignette and survey set, before the first picture and narrative, the following brief instruction was given to participants: “Please, after looking at the picture and reading the text, answer each question ranging from 1 to 6, meaning 1= Absolutely NOT and 6= Absolutely YES. (1 is Absolutely NOT; 2 is Generally NOT; 3 is Somewhat NOT; 4 is Somewhat YES; 5 is Generally YES; 6 is Absolutely YES)”. After every vignette, the next four questions about the interest of the female participants and of their female friends to hook up or have a relationship with the young man described in the preceding vignette followed:

1. Would your female friends like to hook up with him at a party?
2. Would your female friends like a relationship with him?
3. Would you like to hook up with him at a party?
4. Would you like a relationship with him?

The participants answered the questions using a Likert scale which ranged from 1 to 6, where 1 meant ‘totally no’ and 6 meant ‘totally yes’.

### Procedure

This study was carried out in accordance with the recommendations of the ethical guidelines of Marie Curie Actions under Horizon 2020. The protocol was approved by the Ethics Committee involved in the evaluation of Marie Curie Actions (H2020 Rules for Participation, Art. 14. Official Journal of the EU 2013). The project is being also carried out based on the British Educational Research Association Ethical Guidelines for Educational Research (2011) and it has the approval of the University Data Protection officer at the University of Cambridge and the Institute of Criminology’s Research Ethics Committee. All participants in the study were informed about the research and gave voluntary written informed consent in accordance with the Declaration of Helsinki [43]. Participants had time to read the consent form and additional explanation was offered when necessary. The information provided in the consent form explained the purpose of the study, the voluntary nature of participation, the possibility to withdraw from the study at any moment without negative consequences in the college education or in any other life domain of the participants, the materials and measures to be used, the procedure to collect the data, and the anonymity and privacy statement.

The researchers went to the four college classes to distribute the information letters and consents, and to administer the vignette test. The consent form included the contact details of the main researcher of the Free Teen Desire research project, the corresponding author of this paper, for any questions or problems. Once the consent form was properly understood and signed, research participants were given a hard copy of the vignette set and had 15 min to complete the questionnaire. The top of the first page of the vignette set included the instructions for participants regarding how to proceed. Once the participants finished completing the task, they returned the paper copy of the vignette set to the researchers in the room.

### Data analysis

Given that in this study we wanted to explore young women’s coerced preferences for men with violent attitudes and behavior and, more particularly, for what type of sexual affective relationships they coercively preferred such men, we focused the analysis only on responses about vignettes including narratives of men with violent attitudes and behavior, that is, vignette 1, vignette 3, vignette 5, vignette 7, vignette 9 and vignette 11. The analysis follows the same logics of grouping that was employed in the vignette sets.

For the analysis of the responses, Likert scale values were categorized as dichotomies of “No” and “Yes”, where values 1 to 3 signified “No”, and values 4 to 6 signified “Yes”. We analyzed all responses -both those for participants’ female friends’ choices and those referring to participants’ own choices. In addition to this, we examined whether participants would report higher coerced preferences for violent men when asked about the coerced preferences of their female friends than when asked about their own preferences. We expected to be easier for the participants to give an answer informing of coerced preference for men with ‘violent’ attitudes and behavior when responding about their friends’ coerced preferences than about their own.

The data were processed using SPSS version 23.

## Results

The data collected revealed that, while only 28.95% of female participants in the study responded that their female friends would prefer a young man with violent attitudes and behavior for a stable relationship, 58.42% of them would prefer that type of men for hooking up. When female participants reported about themselves, just 5.78% said that they would prefer a young man with violent behavior for a stable relationship, and 28.42% would do so for hooking up. Thus, participants in the study responded both for female friends and for themselves, that they preferred a man with violent behavior more for a hooking up than for a stable relationship (see supplementary material 2. group 1 dataset, supplementary material 3. group 2 dataset, supplementary material 4. group 3 dataset, and supplementary material 5. group 4 dataset).

In what follows, we present these results disaggregating the data according to the responses to the questions for each of the four groups of participants and for the two types of sexual affective relationships (hooking up versus stable) related to men with violent behaviors (See Table 3).

**Table 3:** Female friends’ preferences toward males with violent behavior to hook up (%)

	Group 1		Group 2		Group 3		Group 4		Total	
Question	No	Yes	No	Yes	No	Yes	No	Yes	No	Yes
Would your female friends like to hook up with him at a party? (Violent profile related to vignettes 1,5 and 9)	75	25	45.45	54.55	45.83	54.17	40.74	59.26	41.58	58.42
Would your female friends like to hook up with him at a party? (Violent profile related to vignettes 3, 7 and 11)	64.4	35.6	65.45	34.55	61,7	38.3	44.44	55.56		

In total, 58.42% of the female participants in the study responded that their female friends would prefer to hook up with men with violent behavior. The results disaggregated by group and by violent profile related to vignettes 1, 5 and 9 showed that more than half of the young women in group 2 (54.55%), group 3 (54.17%) and group 4 (59.26%) said that their female friends would prefer to hook up with these men. The narratives about behavior provided in these first violent profiles (manifested in vignettes 1, 5 and 9) described the young men in the following way:

Narrative 1 (Vignette 1 and 5): *He is rough around the edges, but his honey colored eyes, his hair style, his dominant masculinity make the girls go mad for him. My friends say he has everything: show-off, controlling, manipulative. He says precisely what they want to hear, and they hang off his every word. I heard him to tell from a girl he hooked up, that she was a slut, but actually she did not leave him alone. In his group of friends, he is the typical one that fails every single exam but is good at sport.*

Narrative 3 (Vignette 9): *He is a funny bastard. He seems disinterested in girls and can’t even remember their names after hooking up with them… In fact, he laughs at his friends who act like that… by that point some girls like to be with him again and he disdains them persistently. He is not as sensible and good as his friends are, but his strong temper makes him somebody interesting to be discovered. Lots have tried to. He is someone to be rescued. He has a difficult personality.*

However, group 1 was the only group in which the percentage of those participants who responded that their friends would prefer to hook up with this type of men decreased. Nonetheless, it was still 25%.

By analyzing the results disaggregated by group for the second violent profiles portrayed (manifested in vignettes 3, 7 and 11), it was observed a more similar response between group 1, group 2 and group 3 (g1 = 35.6%; g2 = 34.55%; g3 = 38.3%), and a substantial raise in the case of group 4, with 55.56% of the participants answering that their female friends would prefer men with violent behaviors for a hook.

This may have indicated the weak descriptive character of the young men’s pictures for these different vignettes, which did not substantially influence female participants’ preferences. The two different narratives associated with vignettes 3, 7 and 11 described the young men in the following terms:

Narrative 2 (Vignette 3 and 7): *He doesn’t care about what people think; he knows how to get what he wants and uses every last trick to get it. Although initially you don’t care he knows at the end you will fall down in his hands and he persistently chases you till he gets you, even though he ends up being seen as a baddie, a tricky, mysterious, maybe even controlling bloke. He laughs at the girls when they try to hook up with him. Nevertheless, if you're daring and he accepts... you could be the one. Even if he makes you cry, it’s worth it.*

Narrative 4 (Vignette 11): *You want not to like him, but his hypnotic eyes will hook you. You will probably be another girl he’s been with, but most are not ashamed to admit they dream to be the one to save him. Despite of the fact that he has touched them without consent they believe they can change him. He's totally the hottest boy. His personality is scary, but it will make you drool.*

Further qualitative research is being performed in the framework of the Free Teen Desire research project to better understand the specific mechanisms related to social processes and attitudes that coerce young female’s preferences in these ways. Yet regarding what concerns us here, what should be noted is the divergence in the responses for female friends’ preferences between the two different possible relationships provided, hooking up and being in a stable relationship (see Table 4). In reviewing the results for each group, it can be observed that when the same study participants were asked if their female friends would prefer for a stable relationship, instead of hooking up, the same men who were described with violent behavior, percentages decreased considerably. Only 28.95% responded that their female friends would prefer a stable relationship with young men with violent behavior.

**Table 4:** Female friends’ preferences toward males with violent behavior for a stable relationship (%)

	Group 1		Group 2		Group 3		Group 4		Total	
Question	No	Yes	No	Yes	No	Yes	No	Yes	No	Yes
Would your female friends like to have a relationship with him? (Violent profile related to vignettes 1, 5 and 9)	96.67	3.33	70.91	29.09	91.67	8.33	74.07	25.93	71.05	28.95
Would your female friends like to have a relationship with him? (Violent profile related to vignettes 3, 7 and 11)	86.67	13.33	78.18	21.82	85,1	14,9	70.37	29.63		

The data clearly showed that when comparing preferences for having a stable relationship versus hooking up with men with violent behavior, the latter type of relationship was higher than the former in all cases. However, Table 4 shows that there is not a steady pattern in the answers, the violent profiles related to vignettes 1, 5 and 9, or the violent profiles related to vignettes 3, 7 and 11. For instance, it should be noted that the lowest percentages among all of the vignettes were in group 1 (3.33%) and group 4 (8.33%), which corresponded to vignettes 1 and 9, respectively. In the first case (vignette 1), the young man was described clearly as exhibiting dominant behavior (“show-off, controlling, manipulative”, etc.*).* In the second case (vignette 2), the man was also depicted as exhibiting dominant behavior, yet with a personality far from what is socially accepted to establish a stable relationship: (…) [he] *rejects them persistently;* [he] *is not sensitive* (…). Future research needs to be conducted in order to better understand these results in a disaggregated form.

### Participants’ preferences

When participants in the study were asked about their own preferences for hooking up, 28.42% reported that they would like to hook up with young men with violent behavior (see Table 5).

**Table 5:** Personal preferences toward males with violent behavior for hooking up (%)

	Group 1		Group 2		Group 3		Group 4		Total	
Question	No	Yes	No	Yes	No	Yes	No	Yes	No	Yes
Would you like to hook up with him at a party? (Violent profile related to vignettes 1,5 and 9)	100	0	70.91	29.09	77.08	22.92	70.37	29.63	71.58	28.42
Would you like to hook up with him at a party? (Violent profile related to vignettes 3, 7 and 11)	83,1	16,9	83.64	16.36	89.4	10.6	92.59	7.41		

It can be observed that, for the same question, there was a divergence between participants’ own reported response and that for their friends (hooking up = 58.42%). This difference suggested that female participants were better able to recognize the preference toward men with violent behavior in their friends to a greater extent than in themselves.

As was the case when study participants responded about their female friends’ preferences for stable relationships, when answering for themselves the question of whether they would like to have a stable relationship with a young man with violent behavior, the rate of positive answers decreased significantly (see Table 6). Only 5.78% of the female study participants responded positively to this question, with some groups having no females who would like to have a relationship with those men.

**Table 6:** Personal preferences toward males with violent behavior for a stable relationship (%)

	Group 1		Group 2		Group 3		Group 4		Total	
Question	No	Yes	No	Yes	No	Yes	No	Yes	No	Yes
Would you like to have a relationship with him? (Violent profile related to vignettes 1, 5 and 9)	100	0	96.36	3.64	100	0	100	0	94.22	5.78
Would you like to have a relationship with him? (Violent profile related to vignettes 3, 7 and 11)	93.33	6.67	92.73	7.27	100	0	96.30	3.70		

However, a key note between results in Tables 5 and 6 is that the same participants that responded that they would prefer to have a hook with boys with violent behaviors, would not like to stablish a stable relationship with those same boys; this applied to the majority of cases. This analysis can be further enriched with qualitative research that explores the ways in which the coercive discourse operates to produce this difference on female participants at the time of preferring one type of men (with violent behavior) or other (men with non-violent behavior) for different types of sexual and intimate relationships (hooks up or stable relationships).

## Discussion

The study reported here adds to the literature on prevention and response to gender-based violence with a new methodological approach, the use of experimental vignette-based methodology (EVM), to explore young women’s sexual-affective preferences. With the use of such method, the investigation was able to show the participant young women liking men with violent behaviour and liking them in a higher extent for a particular type of sexual-affective relationship: hooking up. Therefore, results point out that hook up becomes a relational context which is potential for violent victimization.

This finding is in line with previous research mainly carried out in the US which pointed out that hooking up is associated with sexual victimization [44, 45]. In this sense, Flack et al. [45] found that students with a history of hooking up were more likely to report incidents of unwanted intercourse. These authors were interested in looking at which types of hookups were riskier for Campus Sexual Assault (CSA). They proved that alcohol consumption and female gender were risk factors for CSA victimization when takes place in the context of hookups. This finding is indeed in line with vast research that evidence that heavy alcohol use can constitute a risk factor for sexual victimization [46, 47]. However, literature has also revealed that college students use alcohol as a tool to facilitate, explain, and justify casual encounters and casual coupling, evidencing thus that alcohol acts as a disinhibiting force and as a justifier of the event at any time when it occurs [48]. Other research has indicated that parties can be sites of offense and violence [49]. Our results add to that research, as hooking up is more common in such spaces.

Being violence in the ‘hookup culture’ at the forefront of the international agenda for gender equality and public health, as well as in the legal field, data reported in this study is of major relevance in order to challenge the dominant public discourse in Spain which solely acknowledges as gender violence the one that occurs within the realm of stable relationships, a public discourse which was reflected in the *2004 Spanish Act against Gender Violence* [24]*.* Considering the reopening of the debate on the law in 2016, our findings contribute to the discussion with evidence supporting the acknowledgment of gender violence beyond stable relationships, such as in dates and hookups and other types of sporadic relationships. If this modification is included in the law, it would have enormous benefits for the victims and for female adolescents generally, as it will be more likely that intervention and prevention programs include a focus, also, on sporadic relationships as potential contexts of gender violence given the existing evidence on dating violence.

Importantly, previous qualitative research on preventive socialization of gender violence has shown that a risk factor for victimization is found in the coercive discourse which, shaped by an imbalance in power within relationships, influences youth socialization into pushing them to feel attraction toward males with violent attitudes and behaviours [50, 51]. According to such prior studies, the coercive discourse can well be acting as a ‘push factor’ affecting the participants’ preferences reported here. Thus, our results also support the need for future research to better understand scientifically the dominant models of male attractiveness which are linked to violence and in which some girls and young women are being socialized.

Data reported in this article also shows that risk factors related to men with violent behaviours and attitudes are quite identified in stable relationships but have not been questioned enough in sporadic ones. Further scientific inquiry could contribute to such line of inquiry by focusing not only on the specific social mechanisms by which such coercive discourse is transmitted to the youth, but also and more importantly, on which interaction processes can be effective in deactivating the coercive discourse. The identification of the later can be part of the social support that many young women need in order to challenge, through their critical consciousness, the coercive discourse that pushes them to a situation of high vulnerability to victimization. In addition, since how violence against women is understood affects how it is treated and prevented, the resulting evidence from those studies could inform intervention and prevention programs that give voice to young women to demonstrate how exciting are the relationships free of violence that they have, whatever format (sporadic or stable) those relationships have.

The results of the study do not ignore the role played by socialization upon men’s sexual-affective behavior. The literature on men and masculinities has accumulated important evidence on how hegemonic masculinity is constructed socially and historically, taught, learn and recreated through many different socialization agents and discourses [33]. In this regard, research has shown that the peer group, depending on the interactions and dialogues among male members regarding gender relations and treatment of women, can promote that some young men scorn women [52].

Our evidence also aids dismantling another assumption widely extended in Spain and which has become a controversial issue, the idea that “ideal love” or the romantic notion of love, associated to stable relationships, is responsible for gender violence in adolescence [26–30]. According to those authors, women are forced to choose in submissive ways for stable relationships of romantic love, which leads women to suffer from violence. This discourse, for example, has presented Shakira as a case of a submissive woman in her stable relationship with Piqué [53], naming Shakira as suffering from the “syndrome of the submissive famous woman” [54]. On the contrary, our findings show that the coercive discourse forces some women to choose in submissive ways for stable, sporadic or hookup relationships with men that have verbal and/or physical violent behavior.

Our data indicated that both when responding according to their own preferences, and when responding according to their friends’ preferences, the participants preferred men with violent behavior fairly more for a hook up than for a stable relationship. On the ground of prior research about preventive socialization of gender violence [5], we hypothesize that preference results from the powerful influence of the coercive discourse in young women’s sexual-affective socialization. In any case, such result counteracts the aforementioned assumption in Spain that blames “ideal love”, associated to stable relationships, for gender violence victimization. This contribution is also relevant at the international level, as the manifold and persisting debates revolving the idea of romantic love does not only exist in Spain but also in other contexts [55]. Our findings go beyond such discussion providing evidence that shed light on the fact that what constitutes a risk factor for sexual victimization is not having a hookup per se or a stable relationship, but if any kind of relationship occurs with someone who can be a perpetrator of violence.

In summary, in the Spanish context that frames the results presented here, two elements must be considered: 1) non-evidence-based campaigns for the prevention of gender violence have emphasized that it is in stable relationships that gender violence occurs. These campaigns are based on the fact that women have been socialized in the search for an “ideal love” that leads them to submit to their partners. If young women are not shown the evidence where it is stated that violence can exist in any type of relationship and that it is not love that causes gender violence but rather choosing violent men to have relationships whether they are sporadic or stable, young women identify that the risk of violence only exists in stable relationships, but not in sporadic relationships or hookups. 2) The message that young women receive from the dominant coercive discourse is that having sporadic relationships with men with violent attitudes and behaviors is what is attractive and exciting. Both elements generate a social pressure that coerces them to have sporadic relationships with this type of man, without considering the risk factors offered by the existing literature. Young women identify that in casual relationships or hookups anything goes because there is no risk. They do not activate the same warning mechanisms that prevent risks when choosing a man for a sporadic relationship or hookup as for a stable one.

There were limitations to this study. The sample of group classes from the two Spanish universities was not randomly selected. Also, the four groups were of students enrolled in education degrees. Therefore, caution should be applied in considering these results, which should not be generalized. Future research could examine young women’s coerced preferences for different types of sexual relationships and for men with different types of sexual-affective behavior (violent and non-violent) in a larger random sample, attending to various international contexts and considering diverse college degrees.

## Conclusions

In the current era of social impact of science [56, 57], medical research needs to advance scientific knowledge about the underlying psycho-social and cultural processes behind all different forms of gender violence victimization (domestic violence, intimate partner violence, dating violence, etc.) to ultimately expand our understanding of this plight in all its forms as well as to better inform policy-making and intervention strategies that reverse the very problematic situation of gender violence worldwide. The fundamental motivation of our study was contributing to such social impact, and thus has important implications for evidence-based prevention and intervention programs in the health and social intervention fields addressed to adolescents and youth. The inclusion of the findings of this study in such interventions can translate into providing female adolescents and young women with some tools to be more critical about the coercive discourse that can push them to coercively prefer men with violent behavior, and thus empower these female youth. Along these lines, the inclusion of the results of this study in gender-violence prevention campaigns and programs in health centers can lead to more effective results in the prevention of violence among young women by means of expanding the types of intimate relationships that are seen and treated as potential contexts of gender violence. Our findings add to current efforts of improving the healthcare response to gender-based violence and sexual abuse [58–60], and can inform preventive campaigns in the media and from health services which reverse the coercive discourse so that what is presented as attractive for young women are violent-free behaviors.

## Supplementary information

**Additional file 1:** Free Teen Desire questionnaire.

**Additional file 2:** Group 1 dataset.

**Additional file 3:** Group 2 dataset.

**Additional file 4:** Group 3 dataset.

**Additional file 5:** Group 4 dataset.

## Data Availability

All data is contained within the manuscript and additional files.

## Abbreviations

ADV: Adolescent dating violence
ARA: Adolescent relationship abuse
CSA: Campus sexual assault
CEDAW: Convention on the elimination of all forms of discrimination against women
EVM: Experimental vignette-based methodology
H2020: Horizon 2020 European Union’s research and innovation programme
IPV: Intimate partner violence
SPSS: Statistical product and service solutions
TDV: Teen dating violence
